# Wnt-5a Signaling Mediates Metaplasticity at Hippocampal CA3–CA1 Synapses in Mice

**DOI:** 10.1007/s10571-024-01512-2

**Published:** 2024-11-13

**Authors:** Jorge Parodi, Rodrigo G. Mira, Marco Fuenzalida, Waldo Cerpa, Felipe G. Serrano, Cheril Tapia-Rojas, Ataulfo Martinez-Torres, Nibaldo C. Inestrosa

**Affiliations:** 1 Centro de Excelencia en Biomedicina de Magallanes (CEBIMA), Escuela de Medicina, Universidad de Magallanes, Punta Arenas, Chile; 2 Departamento de Biología Celular y Molecular, Facultad de Ciencias Biológicas, Pontificia Universidad Católica de Chile, Santiago, Chile; 3 Departamento de Análisis de Datos, Facultad de Ciencias Sociales, Universidad Autónoma de Chile, Temuco, Chile; 4 Centro de Neurobiología y Fisiopatología Integrativa (CENFI), Instituto de Fisiología, Universidad de Valparaíso, Valparaíso, Millenium Nucleus of Neuroepigenetics and Plasticity (EpiNeuro), Santiago, Chile; 5 Centro Científico y Tecnológico de Excelencia Ciencia & Vida, Fundación Ciencia & Vida, Centro de Biología Celular y Biomedicina (CEBICEM), Facultad de Medicina y Ciencia, Universidad San Sebastián, Santiago, Chile; 6 Departamento de Neurobiología Celular y Molecular, Instituto de Neurobiología, Universidad Nacional Autónoma de México, Querétaro, Mexico

**Keywords:** Noncanonical Wnt signaling, Wnt-5a, Calcium, Metaplasticity, Hippocampus

## Abstract

Wnt signaling plays a role in synaptic plasticity, but the specific cellular events and molecular components involved in Wnt signaling-mediated synaptic plasticity are not well defined. Here, we report a change in the threshold required to induce synaptic plasticity that facilitates the induction of long-term potentiation (LTP) and inhibits the induction of long-term depression (LTD) during brief exposure to the noncanonical ligand Wnt-5a. Both effects are related to the metaplastic switch of hippocampal CA3–CA1 synaptic transmission, a complex mechanism underlying the regulation of the threshold required to induce synaptic plasticity and of synaptic efficacy. We observed an early increase in the amplitude of field excitatory postsynaptic potentials (fEPSPs) that persisted over time, including after washout. The first phase involves an increase in the fEPSP amplitude that is required to trigger a spontaneous second phase that depends on Jun N-terminal kinase (JNK) and N-methyl D-aspartate receptor (NMDAR) activity. These changes are prevented by treatment with secreted frizzled-related protein 2 (sFRP-2), an endogenous antagonist of Wnt ligands. Here, we demonstrate the contribution of Wnt-5a signaling to a process associated with metaplasticity at CA3–CA1 synapses that favors LTP over LTD.

**Graphical Abstract:**

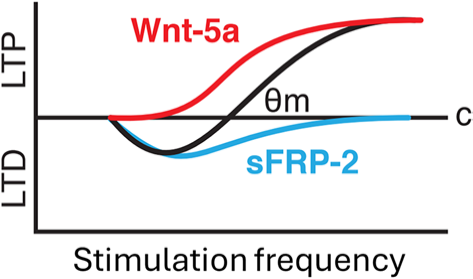

**Supplementary Information:**

The online version contains supplementary material available at 10.1007/s10571-024-01512-2.

## Introduction

Learning and memory depend on an increase or decrease in synaptic efficacy in the central nervous system (CNS), whose magnitude and direction can depend on previous history of synaptic transmission. This phenomenon, called “metaplasticity” (Abraham and Bear 1996), contributes to adjusting the efficacy of synaptic connections to shape network activity, network behavior (Le Ray et al. 2004), and cognitive processes, including learning and memory (Hulme et al. 2013). Certain synaptic proteins, such as postsynaptic density protein-95 (PSD-95), calcium calmodulin kinase II (CaMKII), protein kinase C (PKC), calcineurin, and proteins associated with the calcium (Ca^2+^)-buffering system, modulate long-term potentiation (LTP) or long-term depression (LTD) through metaplastic changes (Jedlicka 2002). The regulation of these proteins and their effects on metaplasticity appears essential for learning because they allow neuronal networks to encode specific information. The existence of diverse molecules that modulate and alter metaplasticity suggests a greater level of complexity of synaptic communication. The molecules that could be important for altering metaplasticity include neurotrophins and factors associated with synaptic maturation (Caroni et al. 2012).

Additionally, certain morphogens, such as proteins of the Wnt family, activate several signaling pathways that play key roles in biological processes from development to adulthood (Nusse and Clevers 2017; Steinhart and Angers 2018). Wnt proteins bind to Frizzled family transmembrane receptors, activating various signaling pathways that regulate a wide range of cellular processes. Wnt proteins could activate the canonical signaling that regulates the β-catenin-mediated transcription of target genes or the noncanonical signaling pathways known as the Wnt/Ca^2+^ pathway or the Planar Cell Polarity (PCP) pathway (Angers and Moon 2009). Wnt signaling plays an important role in the CNS by mediating changes in the connectivity and structure of synapses (Cerpa et al. 2011; Chen et al. 2006; Cuitino et al. 2010; Inestrosa and Arenas 2010; Inestrosa and Varela-Nallar 2014, 2015). Previously, we showed that Wnt-5a, a noncanonical ligand, can increase the amplitude of field excitatory postsynaptic potentials (fEPSPs) in the hippocampal CA1 region (Varela-Nallar et al. 2010) by increasing postsynaptic calcium concentrations, activating PKC and c-jun N-terminal kinase (JNK), and promoting α-amino-3-hydroxy-5-methyl-4-isoxazolepropionic acid receptor (AMPAR) and N-methyl-D-aspartate receptor (NMDAR) currents (Cerpa et al. 2011). These events are correlated with the maturation of glutamatergic transmission and the development of dendritic processes in hippocampal neurons in culture (Farias et al. 2010). Wnt-5a induces a long-lasting increase in synaptic transmission, which is associated with an increase in NMDAR currents (Cerpa et al. 2011). However, whether the components of the Wnt-5a signaling pathway and their downstream effectors (Inestrosa and Arenas 2010; Oliva et al. 2018) are involved in metaplasticity is unknown. Given that various components regulated by the Wnt pathway are also recognized to participate in cellular processes involved in metaplasticity, we hypothesize that noncanonical Wnt signaling may play a role in the regulation of metaplastic events in the hippocampus.

To test this hypothesis, we evaluated whether short-term application of Wnt-5a can modify the threshold required to induce long-term plasticity in the CA1 region. We observed that Wnt-5a reduces the threshold required to induce LTP and inhibits the induction of LTD. Additionally, in the dorsal hippocampus of animals subjected to the Morris water maze (MWM) test, the levels of Wnt-5a increase, whereas the levels of the Wnt signaling inhibitor soluble Frizzled-related protein 2 (sFRP-2) decrease. These findings suggest that the activation of Wnt-5a signaling acts as a metaplastic regulator of the LTP/LTD threshold.

## Results

### The Wnt-5a-Induced Change in fEPSPs in the CA1 Region is Time-Dependent

**Fig. 1:**
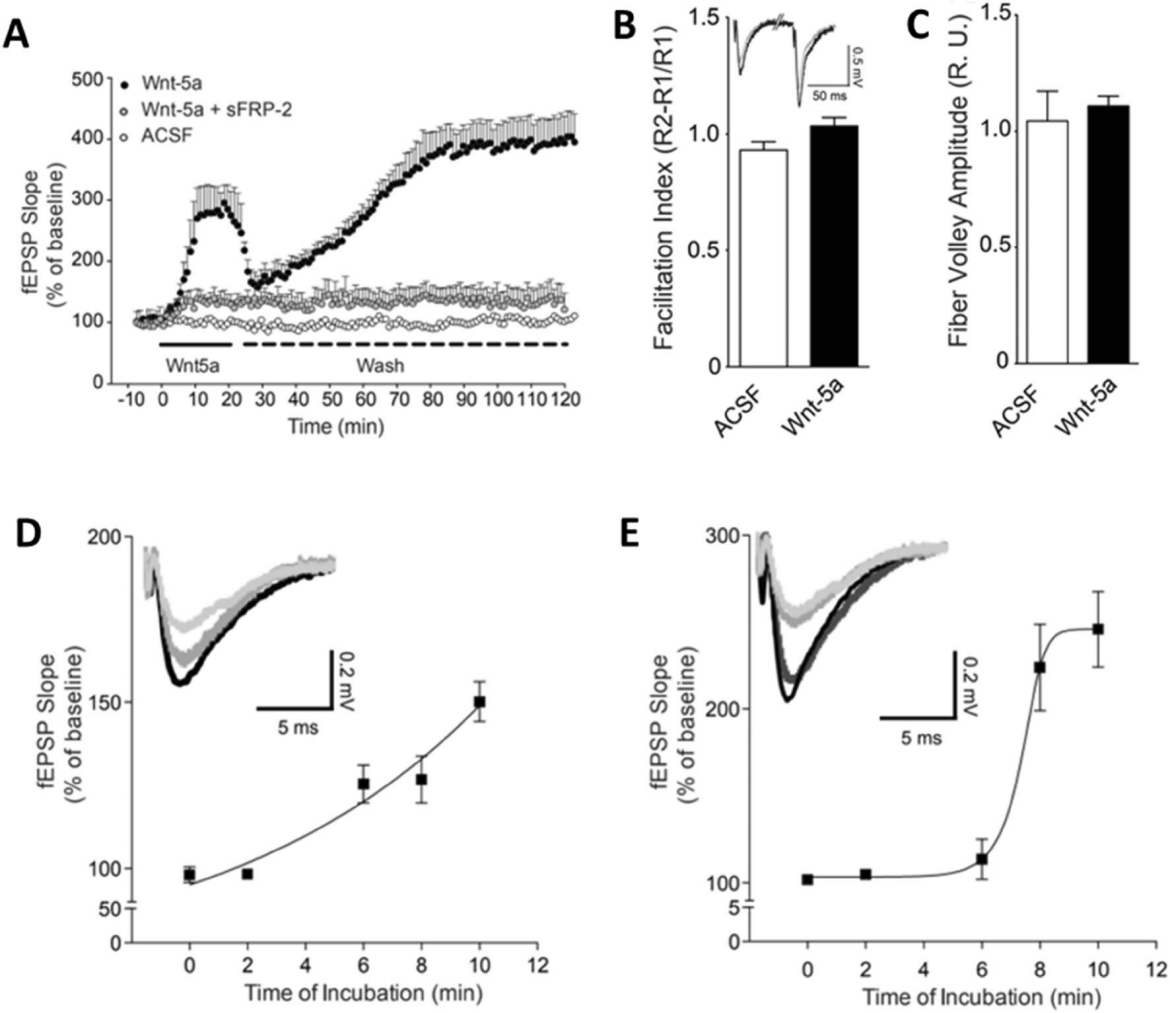
Wnt-5a generates a first response that induces a spontaneous second response. **A** Hippocampal slices were exposed to culture media containing Wnt-5a (2 pM) with or without sFRP-2 recombinant protein (25 nM) for 10 min. The plot shows the change in the fEPSP slope during 10 min of exposure to Wnt-5a and Wnt-5a and sFRP-2 and after 100 min following washout and treatment with or without sFRP-2. **B** Quantification of paired-pulse facilitation in slices exposed to Wnt-5a following the application of two consecutive stimuli (50 ms paired-pulse interval). The inset shows representative traces. **C** Quantification of the fiber volley amplitude in control- and Wnt-5a-exposed slices. **D** Plot showing the first change in the fEPSP slope at different time points during incubation. The samples were treated with the control (time = 0) and Wnt-5a (time = 2, 6, 8, and 10 min). **E** Plot showing the second change (60 min) in the fEPSP slope after different incubation times. The samples were treated with the control (time = 0) and Wnt-5a (time = 2, 6, 8, and 10 min). The bars and dots are the means ± SEs for 6 different independent slices. Student’s t tests were performed, and the asterisks indicate *p* < 0.05

We first evaluated the contribution of Wnt signaling to basal synaptic transmission after a brief application of culture media containing Wnt-5a protein (hereafter just Wnt-5a). Culture media from WT cells was used as the control in all the experiments. We recorded fEPSPs at 20 min after baseline (only the last 10 min are shown in the figures) and after 20 min of exposure to Wnt-5a 2 pM final concentration in ACSF, similar to previous reports (Varela-Nallar et al. 2010; Parodi et al. 2015). In general, a consistent early increase in the fEPSP amplitude was observed in the presence of Wnt-5a (Fig. 1A, black circles) and not present in the control condition or co-incubating Wnt-5a with the Wnt scavenger sFRP-2 (Kawano and Kypta 2003; Ladher et al. 2000). After removing Wnt-5a from the bath, a long-lasting second phase of the fEPSP response was observed (Fig. 1A, black circles), suggesting that Wnt-5a exerted a postsynaptic effect to modulate plasticity since Wnt-5a elicited neither a presynaptic effect, as evaluated by paired-pulse facilitation (Fig. 1B), nor changes in axonal excitability, as evaluated by the fiber volley amplitude (Fig. 1C). To determine the minimal duration of exposure to Wnt-5a necessary to induce the second response and establish a correlation between the first and second phases of the response, we exposed slices to Wnt-5a for shorter periods (2–10 min) (Fig. 1D, E). The exposure to Wnt-5a for 2 min was not able to induce the first response, while after 6 min of exposure, the first response was present (Fig. 1D). When the slices were exposed to Wnt-5a for 2–6 min, the second change in fEPSPs was not observed (Fig. 1E). Nonetheless, upon exposure to Wnt-5a for 8–10 min, the second response was consistently induced (Fig. 1E). This evidence suggests a relationship between the two response phases, with the first phase being necessary to induce the second.

### Wnt-5a Alters Metaplasticity in the CA1 Region by Regulating the Induction and Inhibition of LTP and LTD

**Fig. 2:**
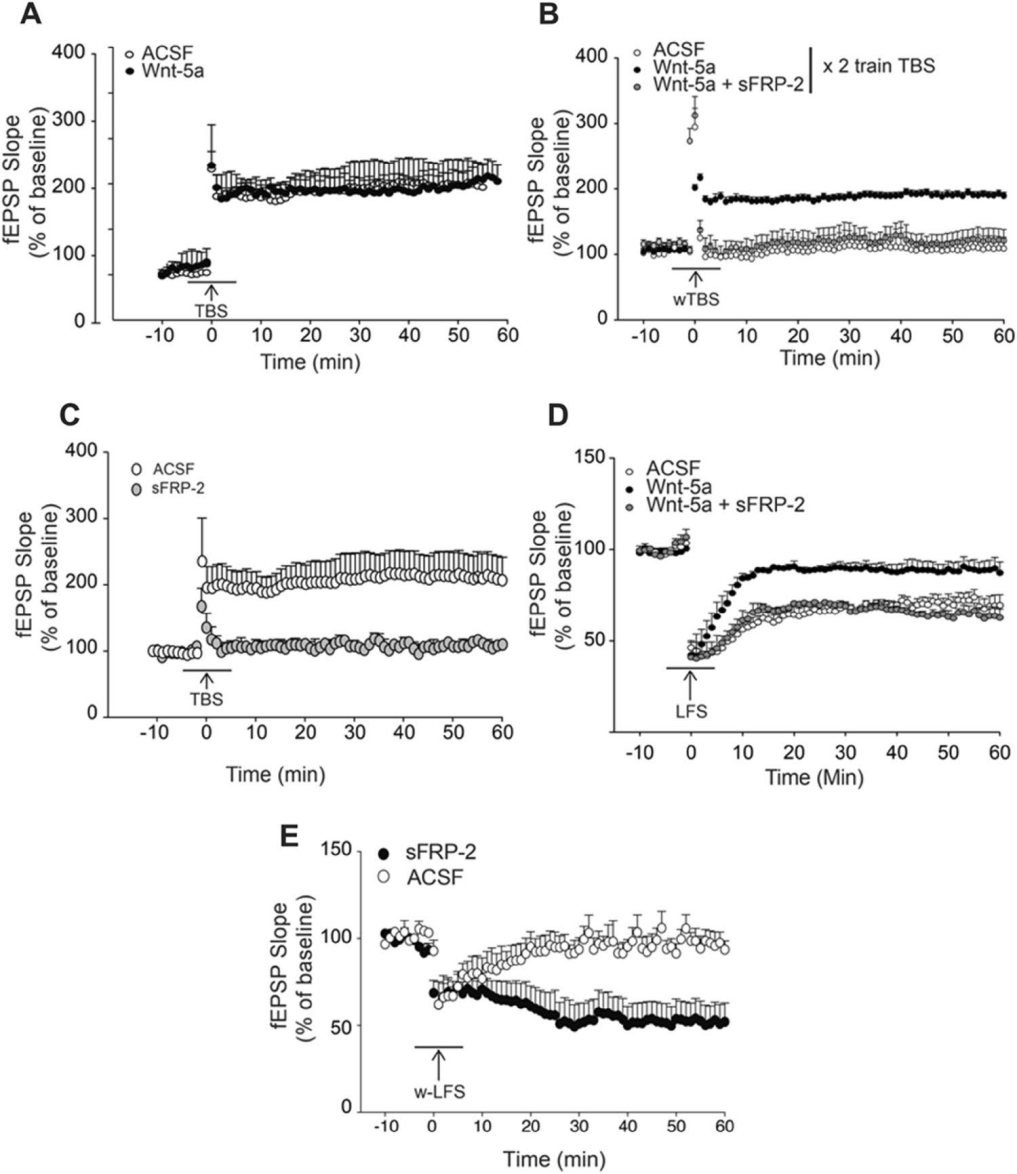
A change in the directionality of plasticity in the presence of Wnt-5a facilitates the induction of LTP and inhibits LTD. **A** Hippocampal slices incubated with the control culture media diluted in artificial cerebrospinal fluid (ACSF) or culture media containing Wnt-5a (2 pM) were subjected to TBS for LTP induction. The arrow indicates time 0, i.e., the time point of TBS, and the black line indicates the time of drug perfusion. The plot shows the change in the fEPSP slope over time. **B** Hippocampal slices incubated with the control culture media diluted in ACSF, culture media containing Wnt-5a (2 pM), or Wnt-5a and sFRP-2 recombinant protein (25 nM) were subjected to wTBS for LTP induction. The plot shows the change in the fEPSP slope over time. **C** Hippocampal slices incubated with the control culture media with or without sFRP-2 recombinant protein (25 nM) were subjected to TBS for LTP induction. The black line indicates the time of drug perfusion. The arrow indicates the time of TBS, and the plot shows the change in the fEPSP slope over time. **D** Hippocampal slices incubated with the control culture media diluted in ACSF, culture media containing Wnt-5a (2 pM), or Wnt-5a and sFRP-2 recombinant protein (25 nM) were subjected to LFS for LTD induction. The arrow indicates time 0, i.e., the time point of LFS; the black line indicates the time of drug perfusion. The plot shows the change in the fEPSP slope over time. **E** Hippocampal slices incubated with the control culture media with or without sFRP-2 recombinant protein (25 nM) were subjected to wLFS for LTD induction. The arrow indicates time 0, i.e., the time point of LFS. The black line indicates the time of drug perfusion. The plot shows the change in the fEPSP slope over time. The dots are the means ± SEs for 7 different slices

Bidirectional long-term plasticity in the form of LTP or LTD is crucial for many aspects of adaptive behavior and brain physiology (Hulme et al. 2013); however, the potential modulatory effects of Wnt ligands on LTP and LTD have not been determined (Oliva et al. 2018). We reason that if Wnt-5a is modifying the basal synaptic transmission, it might facilitate the induction of long-term plasticity. To test this hypothesis, we evaluated the role of Wnt-5a signaling in synaptic efficacy and plasticity at CA3–CA1 synapses in the hippocampus. We evaluated fEPSPs after the induction of LTP to determine whether the activation of noncanonical Wnt-5a signaling can facilitate the induction of LTP at excitatory synapses. There were no changes in the magnitude of LTP in the group incubated with Wnt-5a for a short time (10 min, from − 5 to 5 min, Fig. 2A) and exposed to a strong TBS program (4 trains) compared to the control group (Fig. 2A). In contrast, the addition of Wnt-5a during wTBS (2 trains) facilitated the induction of LTP, an effect that was not observed under control conditions (Fig. 2B, white circles). Wnt-5a facilitated LTP over time, indicating that short-term exposure to Wnt-5a is sufficient to facilitate LTP induction and maintenance in the presence of wTBS (Fig. 2B, black circles). In the presence of Wnt-5a, wTBS-induced LTP was completely prevented by coincubation with sFRP-2 (Varela-Nallar et al. 2010) (Fig. 2B, gray circles). Additionally, we observed that incubation with sFRP-2 alone blocked LTP induction by TBS (Fig. 2C), which is consistent with a previous report (Cerpa et al. 2011). These results strongly suggest a role for Wnt-5a and probably sFRP-2 in synaptic plasticity in the hippocampus.

The role of Wnt-5a and sFRP-2 in the regulation of LTD was evaluated by the application of an LFS program that normally induces LTD at CA3–CA1 synapses (Kemp et al. 2000). This process, as well as LTP, depends on NMDARs and metabotropic glutamate receptors (Citri and Malenka 2008; Collingridge et al. 2010). We observed that perfusion of Wnt-5a inhibited the induction of LTD (Fig. 2D, black circles), whereas coapplication of Wnt-5a with sFRP-2 prevented the inhibition of LTD triggered by Wnt-5a (Fig. 2D, gray circles). Furthermore, in the presence of sFRP-2, wLFS induced LTD (Fig. 2E). These data suggest that Wnt-5a increases the threshold required to induce LTD at CA3–CA1 synapses, whereas sFRP-2 apparently facilitates the induction of LTD at hippocampal synapses.

**Fig. 3:**
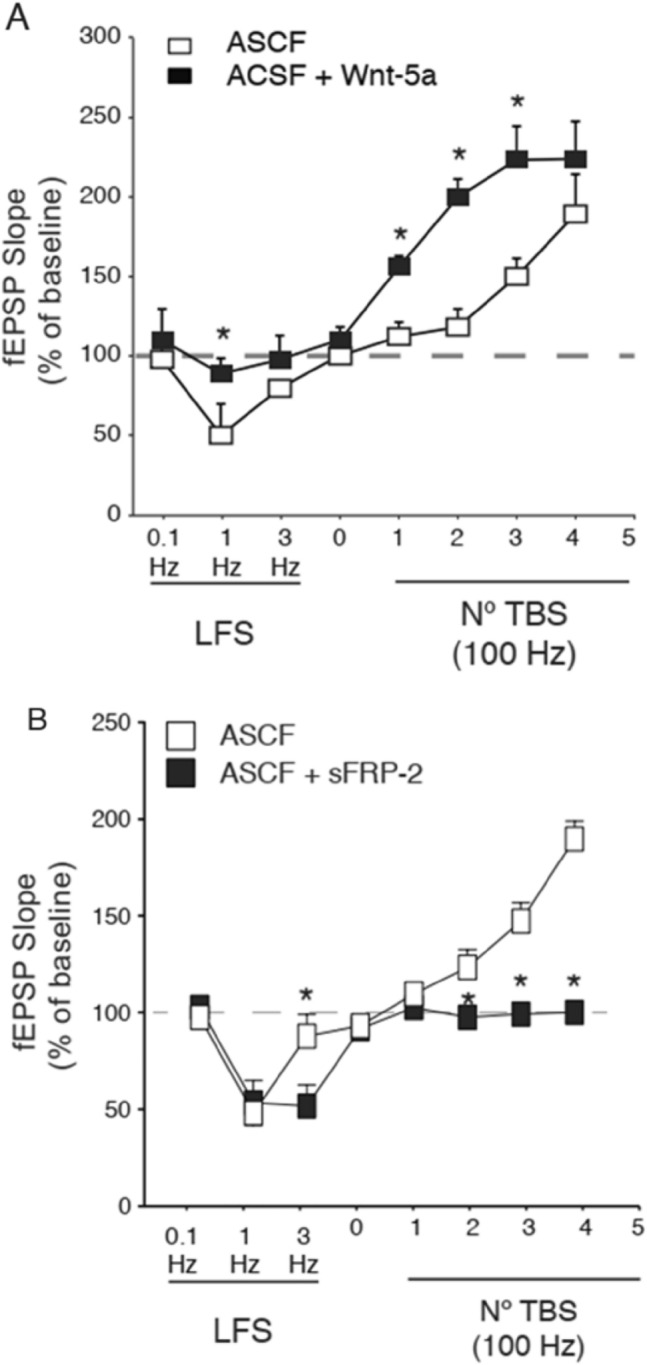
Wnt-5a induces a metaplastic change at CA3–CA1 synapses. **A** Hippocampal slices incubated with the control culture media diluted in ACSF or culture media containing Wnt-5a (2 pM) were subjected to LFS or TBS of different intensities to trigger LTD or LTP, respectively. The fEPSP slopes were analyzed 30 min after LTD or LTP induction. **B** Hippocampal slices incubated with the control culture media with or without sFRP-2 recombinant protein (25 nM) were subjected to LFS or TBS of different intensities to trigger LTD or LTP, respectively. The fEPSP slopes were analyzed 30 min after LTD or LTP induction. The squares represent the means ± SEs for 6 different independent slices. Student’s t tests were performed, and the asterisks indicate *p* < 0.05

The effects of Wnt-5a and sFRP-2 on LTP and LTD could be associated with changes in synaptic efficacy, and to assess changes in the magnitude of fEPSPs, we carried out experiments in which LTP or LTD was induced by different protocols. Short-term application of Wnt-5a altered the threshold required to induce synaptic plasticity, favoring the induction of LTP over LTD (Fig. 3A, black squares). Thus, Wnt-5a generated a metaplastic change in hippocampal transmission. Additionally, sFRP-2 facilitated the induction of LTD (Fig. 3B, black squares), indicating that the balance between the Wnt ligand and Wnt antagonist changed the threshold required to induce synaptic plasticity. The effect of sFRP-2 in promoting the transition from the LTP to LTD indicates a change in the balance of synaptic plasticity in the CA3–CA1 circuit.

### The Effects Induced by Wnt-5a Depend on NMDARs and JNK

**Fig. 4:**
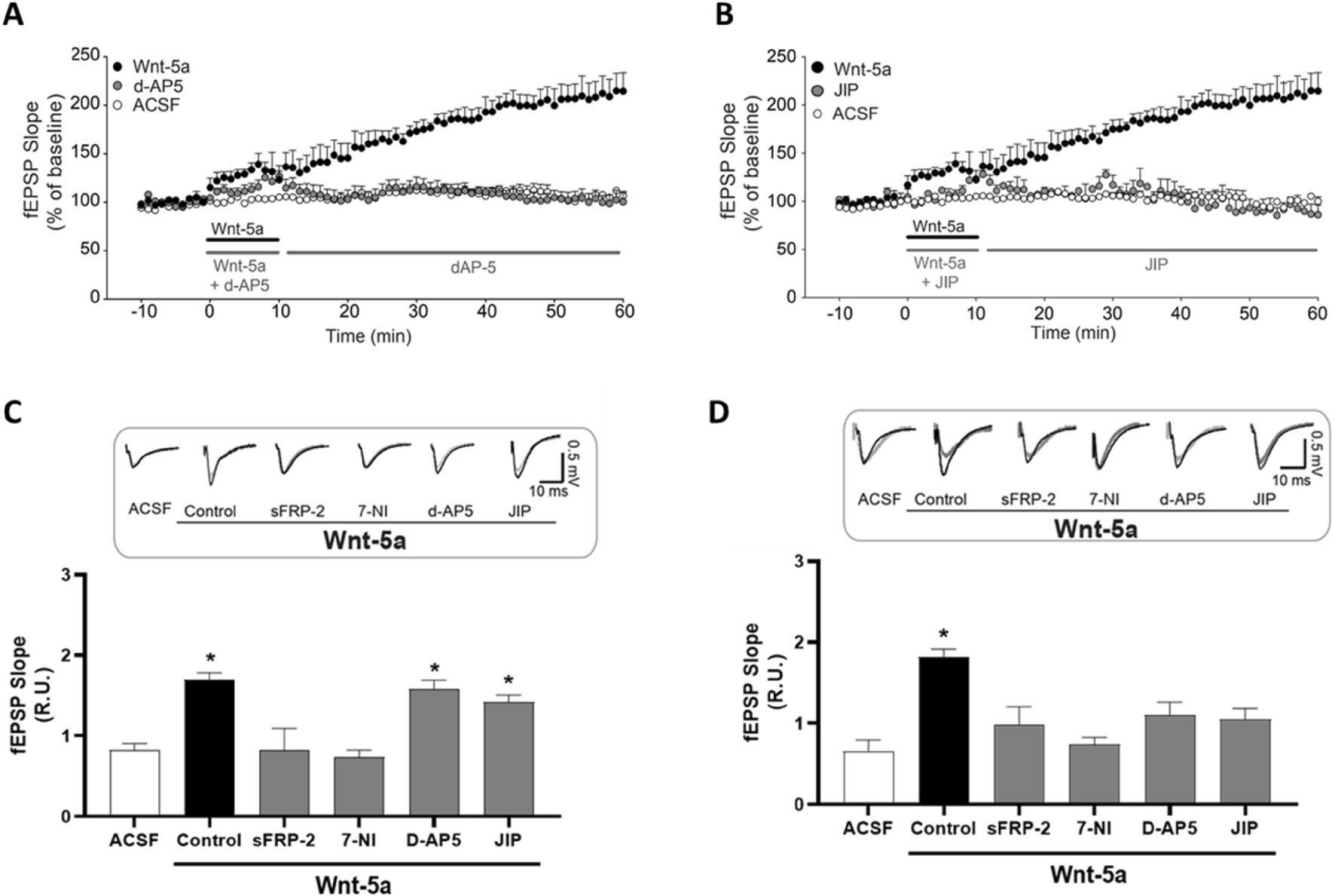
Wnt-5a generates a spontaneous JNK- and NMDAR-dependent second response. **A** The plot shows the change in the fEPSP slope during 10 min of exposure to control culture media diluted in ACSF, culture media containing Wnt-5a with or without d-AP5, and after 40 min following washout and treatment with or without d-AP5. **B** The plot shows the change in the fEPSP slope during 10 min of exposure to Wnt-5a and Wnt-5a plus JIP and after 40 min following washout and treatment with or without JIP. **C** Quantification of the 1st change in the fEPSP slope in the presence of Wnt-5a and sFRP-2,7-NI, d-AP5, or JIP. The inset shows representative traces. **D** Quantification of the 2nd change in the fEPSP slope after 40 min following washout of the ligand and treatment with dFRP-2,7-N, d-AP5, or JIP. The inset shows representative traces. The bars are the means ± SEs for 6 different independent slices. Student’s t tests were performed, and the asterisks indicate *p* < 0.05

We have shown that Wnt-5a increases the amplitude of NMDAR-mediated currents and the LTP magnitude (Cerpa et al. 2011), and a second report indicated possible feedback between Wnt-5a and NMDAR signaling (Li et al. 2012). To evaluate the contribution of NMDARs to the signaling responses to Wnt-5a, we incubated slices with Wnt-5a alone or in combination with d-AP5 (50 μM), an NMDAR antagonist. We did not observe a contribution of NMDARs to the first phase of the response to Wnt-5a (Fig. 4A, gray circles, and 4C), as we previously described (Parodi et al. 2015). On the other hand, after Wnt-5a washout, in the absence of d-AP5, the fEPSP slope increased constantly, representing the second phase of the response to Wnt-5a treatment. In contrast, after Wnt-5a washout in the presence of d-AP5, the second change in fEPSPs was blocked (Fig. 4A, gray circles, and Fig. 4D), suggesting that NMDARs contribute to the effects of Wnt-5a in a later stage of the signaling process.

Another component of the Wnt-5a signaling pathway is JNK, which actively participates in regulating the structure of dendritic spines (Farias et al. 2010; Budnik and Salinas 2011; Farias et al. 2009) and cognitive functions (Pavlowsky et al. 2012). Moreover, JNK contributes to the trafficking of structural proteins and receptors to the postsynaptic density, promoting synapse maturation (Cerpa et al. 2011; Farias et al. 2009, 2010; Cuitino et al. 2010). We evaluated the role of JNK in the time-dependent response elicited by Wnt-5a. We observed that under brief exposure to Wnt-5a in the presence of the JNK inhibitor TAT-TI-JIP (JIP) (Farias et al. 2009), the fEPSP slope was not altered (Fig. 4B). Nevertheless, similar to the effect observed when NMDARs were blocked, the second phase was prevented by JIP (Fig. 4D). To confirm the effects of Wnt-5a on the fEPSP slope, we compared the effects of Wnt-5a with those of sFRP-2 and 7-nitroindazole (7-NI), an inhibitor of neuronal nitric oxide synthase (nNOS; Fig. 4C, D). sFRP-2 avoids both the first and second responses elicited by Wnt-5a given its role as a Wnt antagonist. On the other hand, 7-NI also avoids the first and second responses elicited by Wnt-5a, similar to our previous reports, where 7-NI blocks the effect of Wnt-5a when co-incubated (Parodi et al. 2015). All these data suggest that Wnt-5a generates a fast response, as evaluated by fEPSP, that could correspond to an acute effect of Wnt-5a. These acute changes induce a second response or later response after 60 min. The results suggest that Wnt-5a generates a rapid change in fEPSPs and a second slow and spontaneous change dependent on JNK and NMDAR activity; this finding is similar to that of our previous studies in which patch-clamp recording was performed in the presence of Wnt-5a in the bath throughout the experiment (Cerpa et al. 2011).

### Spatial Learning Induces an Increase in Wnt-5a Levels and a Reduction in sFRP-2 Levels

**Fig. 5:**
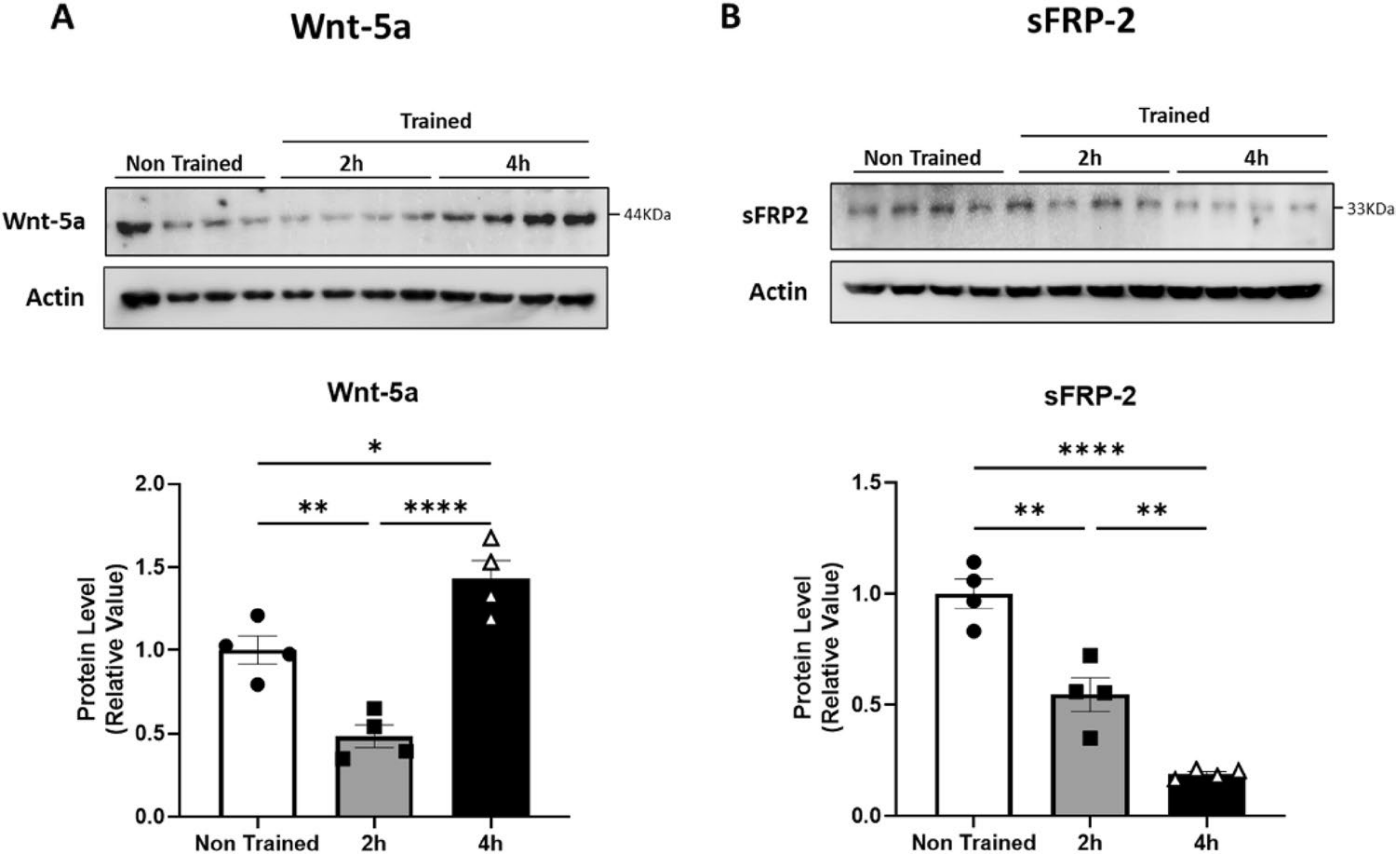
Wnt-5a expression increases after a hippocampus-dependent memory task. **A** Immunoblots showing the levels of Wnt-5a in the dorsal hippocampus of 2-month-old trained and untrained mice. **B** Immunoblots showing the levels of sFRP-2 in the dorsal hippocampus of 2-month-old trained and untrained mice. The graph shows the densitometric value of each protein normalized against to that of the β-tubulin band and compared to the densitometric value of the same protein band in control mice. One-way ANOVA followed by Tukey’s post hoc test. *n* = 4, *p < 0.05; **p < 0.01; *** *p* < 0.001

We evaluated whether Wnt-5a and sFRP-2 influence spatial memory through a modified version of the MWM test [testing memory flexibility (MF)] (Toledo and Inestrosa 2010). During MF the platform location will be successively changed. The platform location will be learned and encoded on memory potentially interfering with the learning that the location of the platform has changed (Chen et al. 2000). Since MF requires both spatial processing and episodic memory (Chen et al. 2000), we suggest that it could be more accurate than classic MWM to resemble a metaplastic change. One set of animals was trained under normal conditions, while another group (control group) was trained in the absence of a hidden platform and spatial cues to prevent spatial information processing. Dorsal hippocampal samples were obtained 2 or 4 h after the completion of the test on the last day of training. MF curves are depicted in Supplementary Fig. 1. Immunoblotting was performed using selective antibodies against Wnt-5a or sFRP-2. Wnt-5a levels increased 4 h after the last day of training in only the trained animals (Fig. 5A), while the levels of the Wnt ligand scavenger sFRP-2 (Cruciat and Niehrs 2013) were reduced in the trained group at both 2 and 4 h after training (Fig. 5B). This suggests that decreased sFRP-2 levels may reduce the probability that the effects of Wnt-5a will be suppressed and eventually facilitate the changes in synaptic plasticity such as the preference for LTP over LTD.

## Discussion

The present work shows for the first time that activation of the Wnt-5a signaling pathway can facilitate the induction of LTP, suggesting that noncanonical Wnt signaling serves as a metaplastic switch that induces long-lasting changes in efficacy of glutamatergic synapses. Metaplasticity, defined as a shift in the threshold required to induce LTP or LTD, along with changes in synaptic efficacy patterns, provides a framework for understanding the intricate processes associated with memory and learning (Abraham 2008; Dunfield and Haas 2009). The effects of Wnt-5a are similar to those observed by other modulators such as brain-derived neurotrophic factor (BDNF), D-serine, or endocannabinoids (Panatier et al. 2006; Rebola et al. 2011; van den Pol 2012; Bortolotto et al. 2005; Edwards et al. 2008; Sajikumar and Korte 2011). These molecules regulate synaptic components (Sajikumar and Korte 2011; Caroni et al. 2012), and hence, metaplastic changes might be linked to events related to the structural dynamics of dendritic spines and processes associated with memory and learning in the hippocampus.

### Wnt-5a-Mediated Metaplasticity

**Fig. 6:**
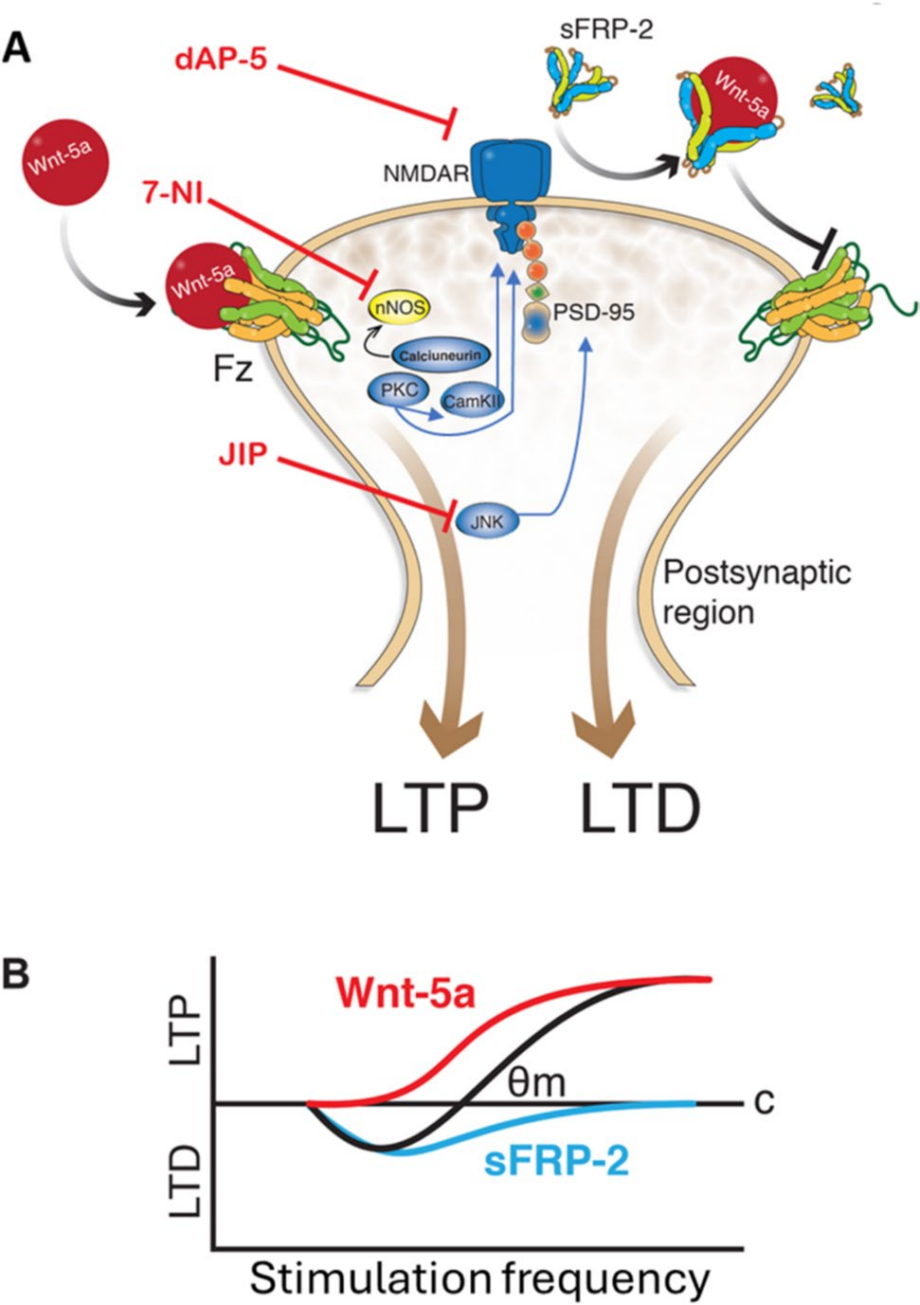
Effects of Wnt-5a on metaplasticity. **A** The mechanism underlying the effects of Wnt-5a involves different events associated with the modulation of metaplasticity by calcium and JNK. **B** Schematic summarizing the roles of Wnt-5a and sFRP-2 in LTP/LTD balance as the synaptic modification function. The black line indicates the normal synaptic modification function with the indicated modification threshold (*θ*_m_). The red line shows the effect of Wnt-5a sliding the *θ*_m_ to the left, and conversely, the blue line shows the effect of sFRP-2 displacing the *θ*_m_ to the right

LTP at glutamatergic synapses is primed by short-term activation of Wnt-5a signaling, which modulates metaplasticity (Fig. 6A, B) to eventually induce a late structural change that depends on proteins such as JNK (Farias et al. 2009). These priming events might depend on the activation of calcium-activated kinases such as CaMKII and PKC, which mediate changes in the induction of LTP at postsynaptic regions (Fig. 6A) (Malinow et al. 1989). Wnt-5a has been widely described to trigger noncanonical signaling through the activation of Frizzled receptors and the recruitment of Disheveled (Dvl), which activate a trimeric G protein. The active G protein, in turn, activates Phospholipase C (PLC), inducing the production of IP_3_ and diacylglycerol (DAG). The release of Ca^2+^ from the Endoplasmic Reticulum (ER) along with DAG activates PKC, but also, the cytoplasmic Ca^2+^ activates CaMKII and Calcineurin (CaN) (Oliva et al. 2018). It has also been described that Wnt-5a induces the activation of JNK through noncanonical signaling (Farias et al. 2009). It is known that in the noncanonical planar cell polarity (PCP) pathway, the activation of Frizzled receptor and Dvl induces the activation of the small GTPase Rac, which, in turn, activates JNK (Oliva et al. 2018; Simons and Mlodzik 2008), despite that, this work and others have not proven this pathway in neurons. Thus, Wnt-5a might activate JNK through Rac1 activation. Importantly, previous studies have indicated that the orphan receptor RoR2 is important for the Wnt-5a-mediated activation of both kinases PKC and JNK (Cerpa et al. 2015), although it has not been demonstrated if only RoR2 or the interaction Ror2-Frizzled receptor is necessary for this activation. Notably, alterations in kinase activity under Wnt-5a treatment have been previously reported (Cerpa et al. 2015). Specifically, investigations using cell culture and reporters of kinase activity based on Förster resonance energy transfer (FRET) signals have indicated that Wnt-5a-mediated activation of PKC occurs more rapidly than Wnt-5a-mediated activation of JNK in the soma and dendrites (Cerpa et al. 2015), indicating an activation temporally differentiated. Interestingly, the levels of PKC are correlated with events of learning and memory in the dorsal hippocampus (Van der Zee et al. 1992; Olds et al. 1989) associated with metaplasticity (Bortolotto and Collingridge 2000), and alterations in synapse structure may give rise to a metaplastic shift, as proposed by Kalantzis and Shouval (Kalantzis and Shouval 2009).

Previous reports have underscored the significance of Wnt ligands in governing both the structure and function of central synapses (Inestrosa and Arenas 2010; Ortiz-Matamoros and Arias 2018; Palomer et al. 2019; Rosso and Inestrosa 2013). It has been suggested that the role of Wnt-5a in synapse remodeling could depend on the exposure time, indicating a specific time course of its effects (Inestrosa and Varela-Nallar 2014; Varela-Nallar et al. 2012). Our studies indicate that Wnt-5a is sufficient to trigger functional modifications that induce a secondary response, promoting changes in the basal state of the neural network and supporting metaplasticity at the synapse, which occurs in a chain reaction similar to the effect of certain neurotrophic factors (Blum and Konnerth 2005; Bramham and Messaoudi 2005; Minichiello 2009; Poo 2001). For example, BDNF induces different patterns of signaling that depend on the duration of incubation, changing the balance of synaptic plasticity (Ji et al. 2010; Kovalchuk et al. 2002; Levine et al. 1998), which is associated with learning and memory (Cirulli et al. 2004). The Wnt-5a response is blocked by sFRP-2, while the second phase of the response is prevented by the application of d-AP5 (NMDAR) or TAT-TI-JIP (JNK) inhibitors. There are several mechanisms by which Wnt-5a induced the activation of NMDARs. The Ca^2+^ released from the ER and the consequent activation of CaN induced the dephosphorylation of nNOS activating the production of nitric oxide (NO) (Munoz et al. 2014). NO increases the trafficking of the GluN2B subunit of the NMDARs to the synaptic surface (Munoz et al. 2014), possibly increasing NMDARs’ content in the synapse. On the other hand, PKC activation has several effects on NMDARs: it increases receptor trafficking to the membrane by sustaining autophosphorylation of CaMKII (Yan et al. 2011), by the phosphorylation of the SNARE protein SNAP-25 (Lan et al. 2001), increasing the channel opening rate (Lan et al. 2001), and activating the interacting protein α2δ-1 (Zhou et al. 2021). These modifications together with the actions of NO on voltage-gated K+ channels (Steinert et al. 2011) and the clustering of PSD-95 mediated by JNK (Farias et al. 2009) lead to a new state of excitability that might account for the metaplastic effect of Wnt-5a. The structural changes mediated by Wnt-5a have been reported in detail using conditional Wnt-5a KO animals. CaMKII-Wnt-5afl/fl mice exhibit a pronounced decrease in dendritic length between 3 and 12 months of age (Chen et al. 2017). This dendritic attrition in CaMKII-Wnt-5afl/fl mice is reversed by the induction of Wnt-5a expression using an adeno-associated virus (AAV) carrying a Cre-dependent Wnt-5a transgene (Chen et al. 2017). Therefore, we propose a dual function of Wnt-5a: *first*, the priming of glutamatergic transmission by calcium, probably through CaMKII and PKC, triggers the facilitation of LTP and inhibition of LTD, a metaplastic process associated with events early in the Wnt-5a signaling pathway; *second*, the late regulation of structural plasticity by JNK and functional activation of NMDARs alter the maintenance of LTP (Cerpa et al. 2011) (Fig. 6A). The potential influence of the late activation of JNK in modulating such changes remains an avenue for exploration; these processes were not evaluated in the current study and will be the focus of future experiments.

Interestingly, other mechanisms could also contribute to the metaplastic changes induced by Wnt-5a in the hippocampus during spatial processing. Wnt-5a is involved in the conversion of silent synapses to functional synapses via NMDAR-dependent and NMDAR-independent mechanisms in neonatal neurons (Alvarez-Ferradas et al. 2022). The unsilencing of synapses has also been suggested to play a potential role in experience-dependent plasticity in the hippocampus in adult animals (Vardalaki et al. 2022; Wang et al. 2019), and it is possible that Wnt-5a might play a role in this process via similar mechanisms. Moreover, the role of NMDARs has not been entirely described and characterized. The use of d-AP5 in this study indicates that the binding of glutamate to NMDARs (Egunlusi and Joubert 2024) is important in the second phase of the Wnt-5a effect. However, the signaling mechanism behind has not been described. NMDARs could signal through ion-dependent mechanisms and ion-independent mechanisms activating different signaling proteins (Park et al. 2022; Dore et al. 2016). The use of channel blockers that do not block glutamate binding, such as MK-801 (Egunlusi and Joubert 2024), would help to elucidate the mechanism of action of NMDARs. Therefore, Wnt-5a could contribute to metaplastic adaptations through different mechanisms, but further investigation is needed.

Importantly, the scavenger sFRP-2, a kind of antagonist, can induce changes in the threshold required to induce synaptic plasticity, modulating the levels of endogenous ligands and possibly altering metaplasticity in the dorsal hippocampus (Fig. 5A, B). This evidence, supported by the facilitation of LTD, implies that the levels of sFRP-2 impact the balance of plasticity decreasing the affinity of Wnt for its receptors (Cruciat and Niehrs 2013), which modulates events such as neurogenesis in the hippocampus (Jang et al. 2013). It is also possible that sFRP plays other modulatory roles, suggesting additional levels of regulation. For example, the negative regulation of certain metalloproteinases, such as ADAM10, by sFRP-1 and sFRP-2 induces the overproduction of amyloid-β (Aβ) peptide, promoting changes in the levels of Aβ (Esteve et al. 2011). Fascinatingly, Aβ has been shown to facilitate synaptic depression akin to LTD (Shankar et al. 2008, 2009), suggesting an additional mechanism regulating long-term plasticity at central synapses with consequences in health and disease.

Additionally, when endogenous Wnt-5a is sequestered with sFRP-2, the synaptic strength of the network is decreased and LTP induction is abolished, despite no change in the induction or maintenance of LTD, which is consistent with the idea that Wnt-5a is present in the hippocampal network and their suppression with sFRPs indicates a putative role of an endogenous Wnt ligand (Varela-Nallar et al. 2010; Gogolla et al. 2009; Cerpa et al. 2010); of course, the removal of the endogenous ligand introduces questions regarding its physiological role and mechanism of action in the hippocampus. Nevertheless, another report indicated that activation of NMDARs induces the synthesis and release of Wnt-5a in neuronal cultures (Li et al. 2012), suggesting the possible existence of a glutamatergic activity-dependent feedback mechanism during signaling that maintains a basal level of the endogenous ligand. The endogenous role of Wnt-5a involves selective modulation of synaptic plasticity, with a certain concentration of the ligand in the region being necessary to modulate synaptic plasticity; this suggests that Wnt-5a also functions like trophic factors, regulating metaplastic changes in the hippocampus.

Therefore, our model proposed that the soluble factors Wnt-5a and sFRP-2 modulate synaptic plasticity changing the threshold required to induce this synaptic plasticity. This could be graphed using the BCM theory (Cooper and Bear 2012), as depicted in Fig. 6B. According to the model proposed by Bienenstock E., Cooper L., and Munro P., neurons possess a modification threshold (*θ*_m_), which determines if a given neuronal activity will strengthen or weaken the synapse, i.e., determine the direction of changes in the synaptic efficacy (Jedlicka 2002). In this model, the metaplastic changes are visualized as the sliding of the modification threshold (*θ*_m_) (Jedlicka 2002). In our results, Wnt-5a decreases the *θ*_m_, decreasing the threshold to induce LTP and preventing LTD. On the other hand, sFRP-2 increases the *θ*_m_, increasing the threshold to induce LTP and facilitating LTD.

### Wnt-5a, Metaplasticity, and Spatial Learning

Our study provides evidence for an increase in Wnt-5a levels within the dorsal hippocampus during spatial learning and memory in the memory flexibility test, as observed during tasks such as the classic MWM test (Tabatadze et al. 2012; Lisman and Otmakhova 2001). Our results, however, did not provide clues about the spatial distribution in the increase of soluble factors Wnt-5a and sFRP-2. The protein levels described here were obtained from hippocampal lysates and thus, they provide total protein content, not only the extracellular pool. Wnt ligands, as secreted glycoproteins, follow the exocytic pathway with complex modifications, including glycosylation and palmitoylation (Mehta et al. 2021; Gross and Boutros 2013). The bands shown in our results correspond to the mature forms of both secreted proteins, given the molecular weight observed. Therefore, the protein levels measured might represent both the secreted pool and the ready-releasable pool. However, to ensure the increase in extracellular levels of both glycoproteins after memory flexibility, protein levels in a microdialysate should be more accurate and remain to be determined.

Metaplasticity has been extensively associated with learning and memory in the hippocampus (Li et al. 2017; Hulme et al. 2013; Xu et al. 2014; Crestani et al. 2019). It is thought that the fact that previous experiences impact learning, either causing interference or promoting a quicker learning process, could be regulated by metaplastic changes in the brain, although the cellular and molecular mechanisms behind them are not entirely understood (Bouton 1993; Tse et al. 2007). It has been described that metaplasticity contributes to memory formation in the hippocampus using the fear conditioning test in a mechanism that involves metabotropic glutamate receptors (Crestani et al. 2019). Moreover, metaplastic changes induced by endocannabinoids are necessary for temporal associative memories but not spatial memory, suggesting that different metaplastic mechanisms might play a role in different forms of memory (Xu et al. 2014). Here, we showed that under a modified version of MWM, the MF test, Wnt-5a levels increased in the dorsal hippocampus, and sFRP-2 levels decreased 4 h after the completion of the task. MF adds an episodic-like component to the classic MWM and hence, the learning and memory processes of one day interfere with the performance of the next day (Chen et al. 2000). Previous reports showed that astrocytic-derived D-serine regulates NMDAR tone which is critical for flexible memory in the MWM test (Koh et al. 2022). In the circuit between Dorsal Raphe Nuclei to the Orbitofrontal Cortex, serotonin regulates reversal learning by regulating excitability, i.e., a metaplastic mechanism (Hyun et al. 2023). While there are no current studies to our knowledge that address metaplasticity and the MF test used here, the evidence showed that cognitive flexibility required in the MF test may involve metaplastic changes through soluble factors, where Wnt-5a and sFRP-2 are good candidates to regulate it.

On the other hand, this finding might also suggest that metaplastic events, regulated by spatial learning and/or cognitive flexibility, are involved in the modulation of the endogenous Wnt-5a levels in the dorsal hippocampus. Previous research has demonstrated a similar phenomenon in the mossy fiber pathway, another hippocampal region, where an enriched environment leads to an increase in the levels of the canonical Wnt-7a ligand (Gogolla et al. 2009) as well as the spatial learning in the MWM test (Tabatadze et al. 2012). Moreover, it has been observed that Wnt-7a is necessary for the consolidation of object recognition memory (Fortress et al. 2013) and it is crucial for maturing synaptic spine structure and enhancing connectivity, particularly during exposure to enriched environments (Caroni et al. 2012). These results suggest that Wnt ligands can regulate synaptic function and structure in the hippocampus during learning and memory. In addition, previous studies using slices from time-dependent Wnt-5a KO (CaMKII-Wnt-5a fl/fl) animals showed a significant reduction in both the induction and maintenance phases of LTP (Chen et al. 2017). Therefore, the presence of high levels of the ligand in the synaptic space for a long period is not necessary for promoting synaptic plasticity.

Our study reveals that Wnt-5a induces metaplastic changes at CA3–CA1 synapses in the hippocampus through the noncanonical Wnt signaling pathway involving NMDAR and JNK in two phases. Moreover, we show that a spatial memory task upregulates Wnt-5a and downregulates sFRP-2 protein expression. This evidence suggests that Wnt-5a participates in processes associated with metaplastic changes through postsynaptic mechanisms during learning and memory.

## Materials and Methods

### Animals and Memory Flexibility Test

Mice strain C57Bl/6J were housed in the Animal Facility of the Pontificia Universidad Católica de Chile under a sanitary barrier in ventilated racks housed 3–5 animals per cage. Animals were maintained at 23 °C in a 12-h:12-h light/dark cycle with food and water ad libitum. The MWM test was performed as previously described by our laboratory (Toledo and Inestrosa 2010). Briefly, 2-month-old C57Bl/6J mice were trained in a circular water maze with a 1.2 m diameter (opaque water, 50 cm deep, 19–21 °C, 9 cm platform placed 1 cm below the water surface, maximum trial duration of 60 s, animals kept for 10 s on the platform at the end of each trial). Each animal was released into the pool from a pseudorandomly selected quadrant and allowed to search for the platform every day for 4 days, and the location of the platform was changed each day. The mice were subjected to training trials until they exhibited an escape latency of < 20 s in 3 successive trials, with a maximum of 10 trials per day. Upon completion of each trial, the mouse was gently removed from the maze and returned to its cage. The animals were trained to find the next platform location on the following day. The animals in the control group were trained within 60 s in the absence of spatial cues and hidden platform for a total of 6 trials per day. The data were collected using a water maze video tracking system for (HVS Image, UK).

### Hippocampal Slice Preparation

Hippocampal slices from 2-month-old C57Bl/6J mice were prepared according to standard procedures (Varela-Nallar et al. 2010). Transverse slices (350 µm) of the dorsal hippocampus were cut in cold artificial cerebrospinal fluid (ACSF, in mM: 124 NaCl, 26 NaHCO_3_, 10 D-glucose, 2.69 KCl, 1.25 KH_2_PO_4_ 2.5 CaCl_2_, 1.3 MgSO_4_, and 2.60 Na_2_HPO_4_) using a vibratome (Leica VT 1000s, Germany), and the samples were incubated in ACSF for more than 1 h at room temperature. The slices were transferred to an experimental chamber (2 ml) superfused (3 ml/min, at 22–26 °C) with gassed ACSF and visualized by transillumination with a binocular stereomicroscope (MSZ-10, Nikon, Melville, NY). The experiments were carried out at room temperature (21–22 °C) in a recording chamber (Carvajal et al. 2018; Cerpa et al. 2015).

### Electrophysiology

Extracellular field excitatory postsynaptic potentials (fEPSPs) of CA1 neurons in the stratum radiatum were recorded, and Schaffer collateral fibers were activated by bipolar electrodes connected to an amplifier (Axon Multiclamp 700b, Molecular Devices, Sunnyvale, CA) and an isolation unit (Isoflex, AMPI, Jerusalem, Israel). A concentric bipolar electrode (platinum/iridium, 125 µm OD diameter, FHC Inc., Bowdoin, ME) was placed in the stratum radiatum 100–200 µm from the recording site. To generate LTP, theta burst stimulation (TBS) consisting of 5 trains of a stimulus with an intertrain interval of 20 s was applied. Each train consisted of 10 bursts at 5 Hz, with each burst containing 4 pulses at 100 Hz. For the weak TBS (wTBS) experiment, we used 2 trains of stimulation. For the TBS intensification experiment, we used 1, 2, 3, or 4 trains. To generate LTD, we used two low-frequency stimulation (LFS) protocols involving 900 paired stimuli (50 ms paired-pulse interval) of 1 Hz (LFS) and 0.1 Hz (wLFS). Recordings were filtered at 2.0–3.0 kHz, sampled at 4.0 kHz using an A/D converter, and stored in pClamp 10 (Molecular Devices). Evoked postsynaptic responses were analyzed offline using analysis software (pClampfit, Molecular Devices), which allowed visual detection of events, and only those events that exceeded an arbitrary threshold were included in the analysis (Carvajal et al. 2018; Cerpa et al. 2015).

### Ligands and Drugs

Control- and Wnt-5a-conditioned media were prepared from L cells (ATCC CRL-2648) and L-Wnt-5a (ATTC CRL-2814) cells (Varela-Nallar et al. 2010). These media were added to ACSF perfusion medium at a final concentration of 2 pM according to a previous report (Varela-Nallar et al. 2010). The mass of Wnt-5a present in the conditioned medium obtained was estimated by immunoblot and used as previously reported for estimated dilution in the ACSF. In all experiments, picrotoxin (PTX) (10 µM, Sigma) was added to the ACSF perfusion medium to suppress inhibitory GABA-A transmission (Cerpa et al. 2011; Cuitino et al. 2010). D(-)-2-Amino-5-phosphono-pentanoic acid (d-AP5, 50 µM, Sigma) was added to the ACSF perfusion medium to suppress NMDAR activity when indicated. Recombinant sFRP-2 (25 nM, R&D Systems) was added with Wnt-5a to the ACSF perfusion medium to block the effect of Wnt-5a. The neuronal nitric oxide synthase (nNOS) inhibitor 7-nitroindazole (7-NI; 1 µM, Sigma) and the JNK inhibitor TAT-TI-JIP (JIP, 1 µM, Merck) were added to the ACS perfusion medium. For immunoblotting, we used anti-sFRP-2 (EPR4832, Abcam) and anti-Wnt-5a (ab72583, Abcam) antibodies.

### Immunoblotting

The hippocampi of the mice were dissected on ice and immediately processed as described previously (Inestrosa et al. 2013; Tapia-Rojas and Inestrosa 2018). Briefly, hippocampal tissues were homogenized in RIPA buffer (10 mM Tris–Cl (pH 4.4), 5 mM EDTA, 1% NP-40, 1% sodium deoxycholate, and 0.1% SDS) supplemented with a protease inhibitor mixture and phosphatase inhibitors (25 mM NaF, 100 mM Na_3_VO_4_ and 30 μM Na_4_P_2_O_7_) using a Potter homogenizer and then passed sequentially through syringes of different calibers. The protein samples were centrifuged at 14,000 rpm at 4 °C twice for 20 min. The protein concentrations were determined using the BCA Protein Assay Kit (Pierce). The samples were resolved by SDS‒PAGE and then transferred to PVDF membranes. Western blot analysis was carried out as previously described (Inestrosa et al. 2013; Tapia-Rojas and Inestrosa 2018; Ramos-Fernandez et al. 2019).

### Statistical Analysis

The initial fEPSP amplitude and slope were measured and normalized to the average baseline value. Data analysis was performed using Prism software (GraphPad Software, Inc.). The results are expressed as the mean ± standard error (SE). The data were analyzed by paired Student’s *t* test or one-way ANOVA followed by Bonferroni’s post hoc test, and *p* < 0.05 was considered to indicate a significant difference. The statistical test used (Student’s t test or ANOVA) is indicated in each figure legend.

## Supplementary Information

Below is the link to the electronic supplementary material.

Supplementary file1 (TIF 70 KB)

## Data Availability

All data supporting the findings of this study are available within the paper and its Supplementary Information. Data details are available upon request to corresponding author via e-mail.
