# Supplementary figures and images for: Wnt-5a Signaling Mediates Metaplasticity at Hippocampal CA3–CA1 Synapses in Mice
Source: Cell Mol Neurobiol. 2024 Nov 13;44:76. doi: 10.1007/s10571-024-01512-2 (PMC11561030; doi:10.1007/s10571-024-01512-2)

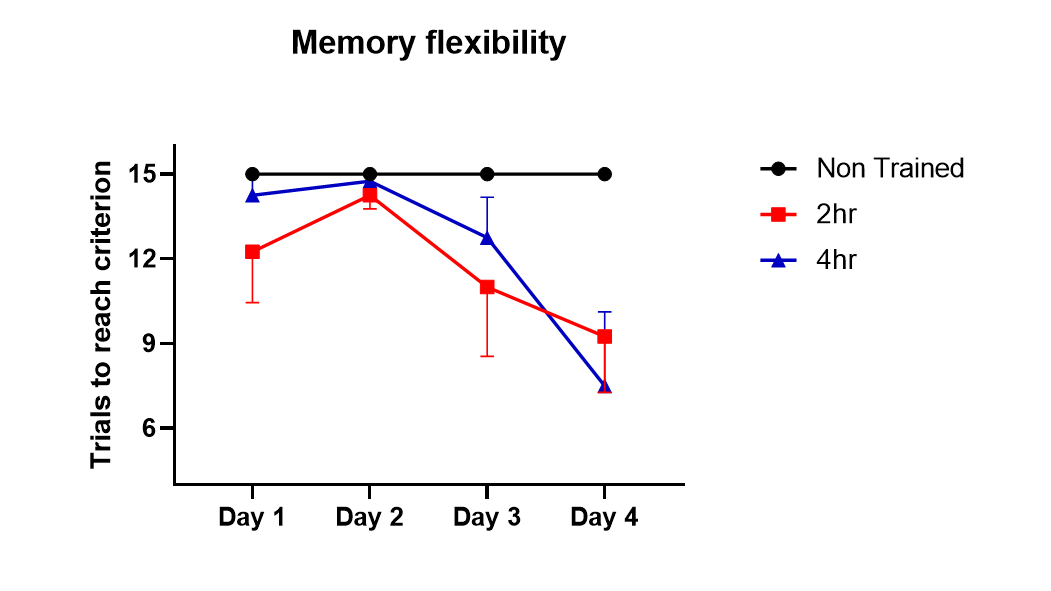

Supplement: Supplementary file 1 — Supplementary file1 (TIF 70 KB) [file 10571_2024_1512_MOESM1_ESM.tif]
